# The modulating effect of food composition on the immune system in growing ring-necked pheasants (*Phasianus colchicus*)

**DOI:** 10.1371/journal.pone.0277236

**Published:** 2022-11-07

**Authors:** Friederike Gethöffer, Jennifer Liebing, Katrin Ronnenberg, Nele Curland, Christina Puff, Peter Wohlsein, Wolfgang Baumgärtner, Bianca Bücking, Ursula Heffels-Redmann, Ulrich Voigt, Christian Sonne, Michael Lierz, Ursula Siebert

**Affiliations:** 1 Institute for Terrestrial and Aquatic Wildlife Research, University of Veterinary Medicine Hannover, Foundation, Hannover, Germany; 2 Thuenen Institute of Biodiversity, Braunschweig, Germany; 3 Department of Pathology, University of Veterinary Medicine Hannover, Foundation, Hannover, Germany; 4 Clinic for Birds, Reptiles, Amphibians and Fish, Justus Liebig University Giessen, Giessen, Germany; 5 Department of Ecoscience, Arctic Research Centre (ARC), Aarhus University, Faculty of Science and Technology, Roskilde, Denmark; University of Life Sciences in Lublin, POLAND

## Abstract

The decline in the population of ring-necked pheasants (*Phasianus colchicus*) in northwestern Germany since 2007 raises questions about the underlying causes. We therefore studied the growth and immune status of ring-necked pheasant chicks dependent on different feed composition. Here, 490 ring-necked pheasant chicks were raised in five groups up to nine weeks. While control groups C1 and C2 received sufficient crude protein (28%) and energy (12.5 MJ/Kg feed) according to current standards, group C2 was treated with cyclosporine eight hours prior to phythemagglutination (PHA) testing, serving as a positive immune suppressed control. Group V1 was fed with reduced protein (20%) but optimal energy content (12.5 MJ/Kg feed), group V2 was fed with sufficient protein (28%) and reduced energy content (10 MJ/kg feed) whereas group V3 was fed reduced crude protein (20%) and reduced energy content (10MJ/kg feed). On all chicks, health status was checked each week, and 20 birds of each group were weighed randomly per week. PHA-testing was performed on 12 birds of each group to study the *in vivo* non-specific activation of lymphocytes at week 2, 4, 6, 7, 8 and 9. In addition, hemolysis–hemagglutination–assay (HHA) was performed on each of the PHA-tested chicks, which were subsequently euthanized and dissected. Histopathologic examinations of 5 birds that were randomly chosen were performed. The PHA–test results demonstrate significant differences between control (C1, C2) and experimental groups (V1-V3) in several developmental stages. According to the HHA results, weekly testing detected a significant increase of titres per week in all groups without significant differences. Here, only hemagglutination and no lysis of samples was observed. It seems appropriate to conclude that during their first weeks of life, protein content is of higher importance in ring-necked pheasant chicks than energy intake. In particular T-cell response is significantly reduced, which indicate a weaker immune system resulting in a higher risk for clinical diseases. Therefore, we assume that protein *i*.*e*. insect availability is a highly important co-factor in the free-ranging population dynamics, and is linked to declines of the northwestern German population.

## Introduction

The ring-necked pheasant (*Phasianus colchicus*) is a ground—breeding and sentinel bird of the agricultural landscape in northwestern Germany. As a breeding bird, the ring-necked pheasant has established in Germany about 200 years ago [1]. It prefers structurally semi—open landscape, where trees and hedges offer sufficient cover, including adjacent sparse forests and reed areas [2].

For several years, a persistent decline of ring-necked pheasants was observed in northwestern Germany [3]. Previous studies on the health status of wild pheasants showed that a large proportion of chicks carry various parasites and pathogens, with especially the young chicks being of low nutritional status [4]. The population achieved a maximum in between 1960 and 1970 and in this period, the hunting bag enfolded about 300.000 pheasants in Lower Saxony [5]. During the hard winters in the 1970ies, the population declined severely, followed by an increase without reaching the former level. Since 2008, the pheasant is again experiencing an immense and sustained decline of unexplained causes and a population reduction of up to 75% has been seen in specific subpopulations [6, 7]. The population density of the hens in the main distribution areas is 8–12 hens/km^2^ while in the wooded areas of north-western Germany the density is at <5 hens/km^2^ [7].

This population decline causes controversial discussions among stakeholders. Above all, the growing amount of agricultural area used for biogas production which extended corn cultivation to about one third of arable land [8], resulted in the loss of adequate habitat. In addition, pesticide use, predation and food availability and composition are important factors in the populations struggle throughout the chick rearing phase [9–12].

The main food composition of adult birds primarily consists of various parts of seeds, berries, tubers, root shoots, leaves as well as green sprouts. In addition, up to 9% of early summer diet is composed of insects, snails and worms [2]. Pheasant chicks, however, live purely insectivorous in the first four weeks of life [2, 13, 14]. It is important that the chicks feed on a variety of foods and can capture a wide range of different insect groups [15]. Protein availability is essential for growth and development of juvenile birds [16], and might also effect immune system performance [17–20].

It has not yet been clarified whether reduced insect availability during rearing of pheasants has secondary effects on the chick immune system, which can consequently lead to an increased susceptibility to diseases and early death of the chicks. Investigations by Curland and others (2018) [3] on the occurrence of free-ranging pheasants showed that the free-ranging population is confronted with a variety of diseases. Studies from England highlighted that partridge chicks, feeding almost exclusively on insects during the first two weeks of life, were dying if those were not abundant [21]. We therefore studied the effects of protein and energy malnourishment in ring-necked pheasant chicks in a classical randomised case-control study. To assess information on immune status of the birds, we used the phythemagglutination test (PHA), in which a skin swelling as a merely T-cell-mediated immune response after injection of phythemagglutinin is measured [22]. It is used by several authors to define immune response in birds, especially if originating from the wild [23–28]. Additionally, hemolysis–hemagglutination–assay (HHA) was applied to figure out natural antibody-mediated complement activation and red blood cell agglutination in wild birds, referring to a single sample of blood only [29] and therefore being applicable for future settings in field testing. We hypothesized that while rearing chick pheasants, a protein deficiency will lead to similar or more severe retardation of growth and immune competence than a low energy diet. Therefore, we expect an effect of protein availability during the first weeks of pheasant chicks’ life on their immune competence, probably leading to a higher susceptibility to diseases and increased mortality.

## Material and methods

### Housing

In total, 490 ring-necked pheasant (*Phasianus colchicus*) chicks were raised from hatching until a maximum age of nine weeks. The chicks were housed in a poultry enclosure on the Farm for Education and Research in Ruthe, University of Veterinary Medicine Hannover Foundation (TiHo). Before entering the enclosure as hatchlings, all birds were tested for Avian Influenza. Every contact with the birds accomplished high—standard hygiene protocols [30]. Cleaning of the enclosure as well as water and food supply and visual health check of all birds were performed twice a day. The temperature and humidity were adapted to the respective recommendations [31], to guarantee an optimal climate during all times of the study. The 490 birds were randomly divided into five groups of 98 each, in which they were randomly equipped with foot rings of seven different colours. Consequently, 72 birds of each group were tested, whereas 26 remained as a reserve. Each group was held in a roofed compartment with a surface area of 36 m^2^ and raised under similar conditions. The compartments were parted by solid metal grids using a mesh of 2×2 cm width. In order to guarantee isolation of the groups, the metal grids additionally were draped with a one meter high black foil. In addition, nets of 2x2 cm mesh width were draped around the compartment to prevent the birds from escaping. Floor covering consisted of a 5–10 cm thick layer of coarse wood chips. During their 1^st^ week of life, chicks were held in rings of 3 m diameter with gas heaters as heat source. After the 1^st^ week, the entire compartment of 36 m^2^ was available. Seat posts, straw balls and balloons were offered as enrichment. Twenty birds of each group were weighed and examined by a veterinarian every week. For PHA and HHA testing, 12 chicks of each group were moved into cages of four m^2^ per group for 24 hours and subsequently euthanized. The experimental design included the euthanasia of the remaining birds at an age of nine weeks. The study was conducted with a permission from the lower Saxony state office for consumer protection and food safety (33.12-42502-04-16/2133).

### Feeding and experimental groups

All groups received an ad libitum feeding and watering. Two control groups (C 1 and C2) received an optimal feed for pheasants with a crude protein content of 28% [31] and 12.5 MJ/Kg of convertible energy (S1 Table in S1 File, [32]), reflecting national standards that differ slightly from international releases [33]. Two control groups were necessary to evaluate the PHA test (see below), group C2 serving as a positive control for immune suppression. Experimental group V1 was fed with low protein content and optimal energy content (20% of crude protein and 12.5 MJ/kg energy). Experimental group V2 was fed with optimal protein content (crude protein content of 28%) and a reduced energy content (10 MJ/kg) while experimental group V3 was fed with reduced protein content (20% crude protein) and reduced energy (10 MJ/kg). With this feeding regime, a reduced insect availability and thus a reduced crude protein content in the diet was simulated in group V1. The feeding of group V2 should facilitate differentiating adequate availability of protein in combination with insufficient energy content, while the nutritional needs of group V3 was both insufficient in protein and energy content. This to clarify if any alterations detected originates from low protein or low energy or only occurred in combination. Feed consumption was noted by weighing the absolute feed weights for each group at the beginning and end of the experiment. All feed compositions were calculated and prepared by the Institute for Animal Nutrition (TiHo).

With this experimental design, we aimed to simulate the potential availability of insects as a main food source in free- ranging pheasant chicks and the possible effects of insufficient protein or energy on growth, health and immune system.

### Phythemagglutination (PHA)—Test

PHA swelling response of the patagial web [22, 24] was measured among the control and experimental groups. After measuring and marking the patagium (mm), a PHA–PBS–mixture (1.0 mg/ml, phytohemagglutinin, PHA-P dissolved in **P**hosphate **B**uffered **S**aline) of 0.1 ml was injected in the right wing patagium, and a negative control of 0.1 ml PBS was injected in the left wing patagium [34]. 24 hours later, both injection sites were measured [mm] again [28]. PHA index was calculated based on the following equation:

$$PHA_{index}=\underline{post‐PHA‐post\;PBS/(pre\;PHA+pre\;PBS)/2}$$

Formula for calculating the PHA index. Pre-PHA: skin thickness before injection with PHA; Post-PHA: skin thickness after injection with PHA; Pre-PBS: skin thickness before injection with PBS; Post-PBS: skin thickness after injection with PBS.

12 birds of each group were randomly sampled by foot ring colour and tests were performed during 2^nd^, 4^th^, 6^th^, 7^th^, 8^th^ and 9^th^ week of life.

Control group C2 served as a positive control for immune suppression. It was treated with 100 mg/kg cyclosporine (for T-cell inhibition) i.m. (Novartis Pharma, Sandimmun^®^ Optoral 10 mg soft capsules) in the pectoral muscle (M. pectoralis) eight hours prior PHA testing and blood sampling. Control group C 1 remained untreated.

### Hemolysis-hemagglutination assay (HHA)–Test

24 hours after PHA–testing, 0.1 ml blood from V. cutanea ulnaris superficialis was sampled in a heparinized capillary tube and kept cool on ice for the next two hours. Thereafter, samples were centrifuged and plasma was frozen at -30° Celsius. HHA–test was performed following protocols of Matson [29] and Wegmann and Smithies [35] to investigate the innate humoral immunity. In short, the assay was performed in 96-well round bottom assay plates. 25 microliters of plasma were pipetted in columns 1 and 2 of the plate. 25 μl of 0.01M phosphate buffered saline were added to columns 2–12. The Plasma in column 2–11 was serially diluted 1:2. 25 μl of a 1% rabbit red blood cell suspension was added to all wells. The plates were sealed with Parafilm and covered with a plate lid. The plates were vortexed carefully and incubated at 37°C for 90 minutes. The plates were tilted to a 45° angle and photographed in front of a light background.

### Histopathological examinations

From 5 randomly selected birds of the groups C1, V1, V2 and V3, immune organs (Bursa fabricii, spleen and thymus), bone marrow, pectoral muscle, skin, heart and liver were collected after euthanasia for histopathological examination. All tissues were fixed in 10% neutral-buffered formalin, embedded in paraffin, wax sliced at a thickness of 3 μm and stained with hematoxylin and eosin (HE) for histological examination according to former studies [3]. In selected cases, Ziehl-Neelsen´s stain, periodic acid Schiff reaction, Brown & Brenn stain and Turnbull blue stain were applied [36, 37].

### Statistical analyses

To test whether body weight gain differs between groups, we tested for group and week along with their interaction. Age in weeks was defined as factor in order to capture non-linear relationships in the chick’s development. Likewise, to test PHA-index in dependence of age and group we also included week as factor and treatment group with their interaction. The overall dependence of body weight and pha-index was tested in a third model, differentiating between the experimental groups. Here, we omitted the data from week two.

Body weight and PHA index were tested for normality (Shapiro-Wilk’s normality test) and homoscedasticity (F test). Residual variance seemed to vary both within age- and group thus several weights structures were tested and the best fit was chosen by AIC comparisons. Accordingly, GLS (generalized least square) in the package nlme was applied to account for heterogeneity in the variances. The best fit of the weights structure was assessed using maximum likelihood estimation in model comparisons including different possible weights structures including week and group for the first two models and chicken weight and group in the third model. The best model by AIC comparisons was refitted using restricted maximum likelihood estimation. For posthoc tests the package emmeans was used using tukey tests. Significance was set to α = 0.05. All statistical analyses were performed using R (R. C. Team, 2019) and RStudio (R. S. Team, 2018).

### Ethics statement

Handling of the animals was conducted under a permit held by the Institute for Consumer Protection and Food Safety of Lower Saxony (Dep. 3 Animal Health, permit number: 33.12-42502-04-16/2133). Animals were euthanized with pentobarbital overdose. All experiments were performed in accordance with institutional and national laws and ethical principles.

## Results

Of 490 chicks, 58 died or had to be euthanized due to severe symptoms of deprivation. Therefore, the average mortality rate was 12%, which is similar [31] or higher [16] to previous studies in ring-necked pheasants. Involved were five chicks (5%) from group C1, 18 chicks (18%) from group C2, 16 chicks (16%) from group V1, nine chicks (9%) from group V2 and ten chicks (10%) from group V3. All deaths occurred before the 7^th^ week of the study, 78% (n = 46) within the first three weeks. In all groups, cannibalism was observed, mainly in group V1 (n = 6), followed by groups V3 (n = 3), C2 (n = 3), C1 and V2 (both n = 1, Table 1). Cannibalism was limited to feather picking on the wings and back, as well as the cloacal tissue.

**Table 1:** Food uptake rate in kilograms, cannibalism and died chicken, split in groups.

Group	Food uptake rate in kg	Cannibalism (n)	mortality (n)
**C1**	50.4	1	5
**C2**	31.6	3	18
**V1**	36.6	6	16
**V2**	80.4	1	9
**V3**	41.6	3	10

### Feeding and body weight development in experimental groups

**Fig 1:**
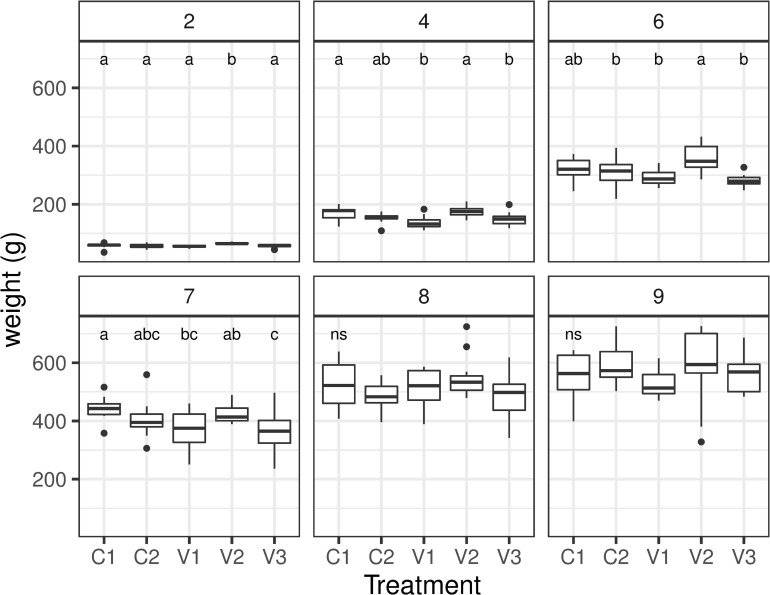
Weight development of the chick groups over the 9 weeks of the study. Small letters indicate significant differences between groups within weeks with 2,4,6,7,8,9 indicating the age of the chickens in weeks.

Overall, the feed consumption of the groups was 50.4 kg in group C1, 31.6 kg in group C2, 36.6 kg in group V1, 80.4 kg in group V2 and 41.6 kg in group V3 (1). Considering the loss of animals in each group within the first two weeks, consumptions per bird were different between groups, with an exceedance of group V2 (Table 1). The body weight development also showed significant differences between groups (Fig 1). While body weight increased in all groups, there were differences in body weight development detected between groups. At the beginning of the experiment in week 2, only group V2 showed a significantly higher body weight. Also, at the end of the experiment in the weeks 8 and 9, no significant differences between groups were detected (S2 Table in S1 File). However, in week 4 and 7, protein depleted treatment group V1 showed significantly lower body weight than the control group C1 (Table 3). In the group fed with reduced protein and energy content V3, mean body weight was significantly lower than in group C1 during weeks 6 and 7 (S2 Table in S1 File). The body weight of the control group C2 showed no significant difference to group C1.

### PHA-test

**Fig 2:**
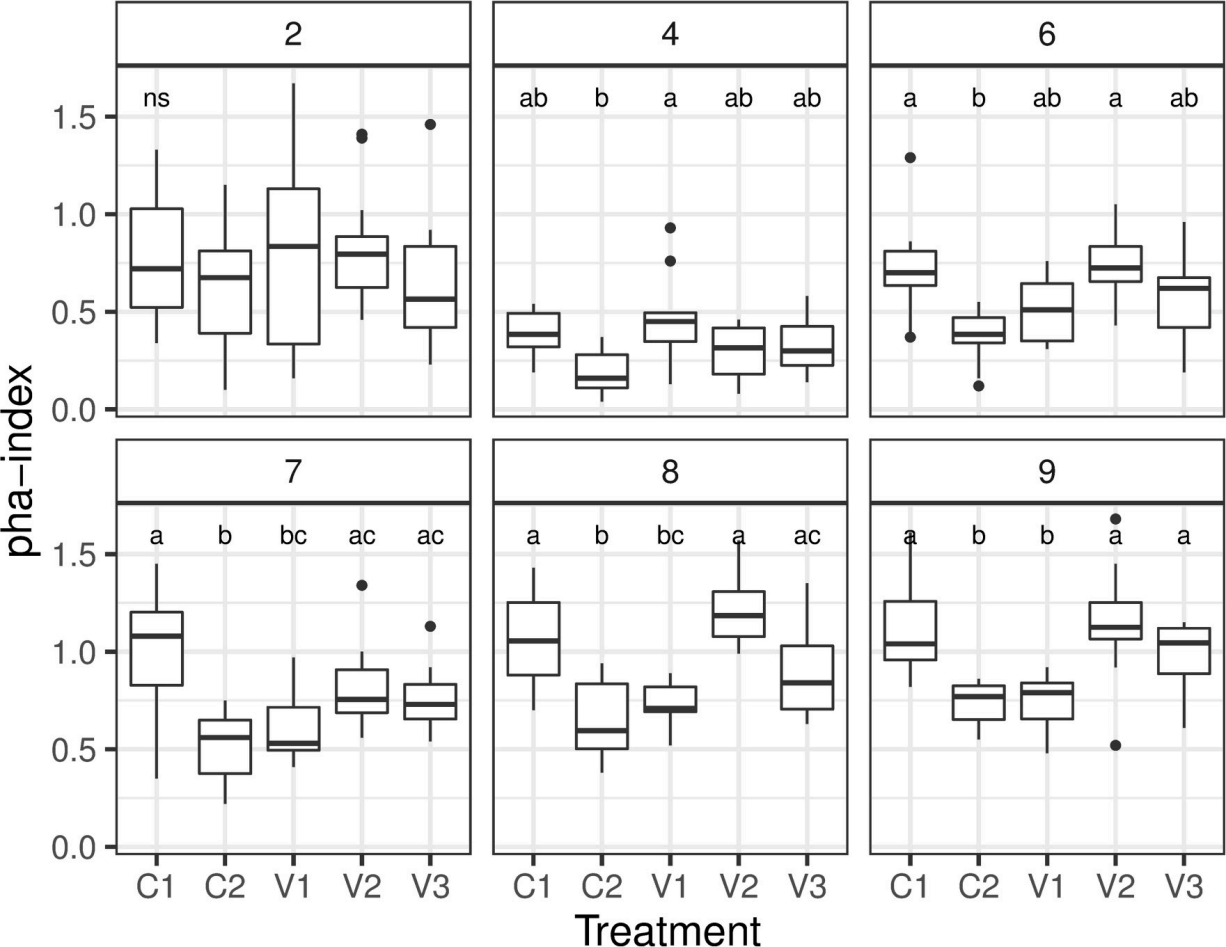
Boxplot presenting the results of the PHA test over the nine weeks of the study. Small letters indicate significant differences between groups within weeks with 2,4,6,7,8,9 indicating the age of the chickens in weeks.

Over the weeks, the PHA-index showed a dynamic pattern. While values in week two were relatively high, they dropped towards week four significantly (Table 2, Fig 2). Thereafter, values of the control group C1 increased steadily and were significantly higher in weeks eight and nine (Table 2). This overall pattern also applied to the other groups, only the protein deprived group V1 showed a slower recovery as shown by significantly lower values for weeks seven and nine (Table 2). Although the overall pattern was comparable for all groups, the Tukey post-hoc test revealed significant differences between treatment groups in several developmental stages. In the cyclosporine treated control (C2), PHA-index was significantly lower from week six onwards compared to C1. The protein deprived group V1 showed a less steep decline in week four but also had significantly lower PHA-indices from week seven onwards compared to C1. The energy deprived group V2 always showed comparable values to the control C1. While V3 showed overall lower mean PHA-indices than C1 and V2, values were never significantly different in the Tukey-test (Fig 2).

**Table 2:** Coefficients of the generalized least square analyses of pha-index dependent on group and age in weeks. The table shows the estimated value, the standard error, the t-value and the significance (p-value) of the terms. The rows represent the groups, the age in weeks and their interactions. Values indicate the differences to the reference value (intercept), which represents pha-index from the control group C1 at the age of two weeks.

	Value	Std.Error	t-value	p-value
(Intercept)	0.779	0.127	6.137	0.000
week 4	-0.390	0.146	-2.674	0.008
week 6	-0.061	0.143	-0.426	0.671
week 7	0.227	0.143	1.586	0.114
week 8	0.282	0.143	1.971	0.050
week 9	0.326	0.143	2.280	0.023
C2	-0.165	0.153	-1.075	0.283
V1	0.013	0.166	0.075	0.940
V2	0.053	0.167	0.319	0.750
V3	-0.145	0.167	-0.867	0.387
week 4: C2	-0.034	0.177	-0.192	0.848
week 6: C2	-0.180	0.174	-1.037	0.300
week 7: C2	-0.321	0.173	-1.852	0.065
week 8: C2	-0.241	0.173	-1.394	0.164
week 9: C2	-0.200	0.174	-1.150	0.251
week 4: V1	0.067	0.191	0.348	0.728
week 6: V1	-0.213	0.188	-1.132	0.259
week 7: V1	-0.398	0.188	-2.120	0.035
week 8: V1	-0.347	0.189	-1.840	0.067
week 9: V1	-0.384	0.188	-2.043	0.042
week 4: V2	-0.137	0.191	-0.721	0.472
week 6: V2	-0.048	0.188	-0.257	0.798
week 7: V2	-0.246	0.188	-1.306	0.193
week 8: V2	0.093	0.188	0.491	0.624
week 9: V2	-0.012	0.188	-0.062	0.951
week 4: V3	0.084	0.191	0.440	0.660
week 6: V3	-0.006	0.189	-0.032	0.974
week 7: V3	-0.104	0.188	-0.553	0.581
week 8: V3	-0.033	0.189	-0.175	0.861
week 9: V3	0.006	0.188	0.031	0.975

**Fig 3:**
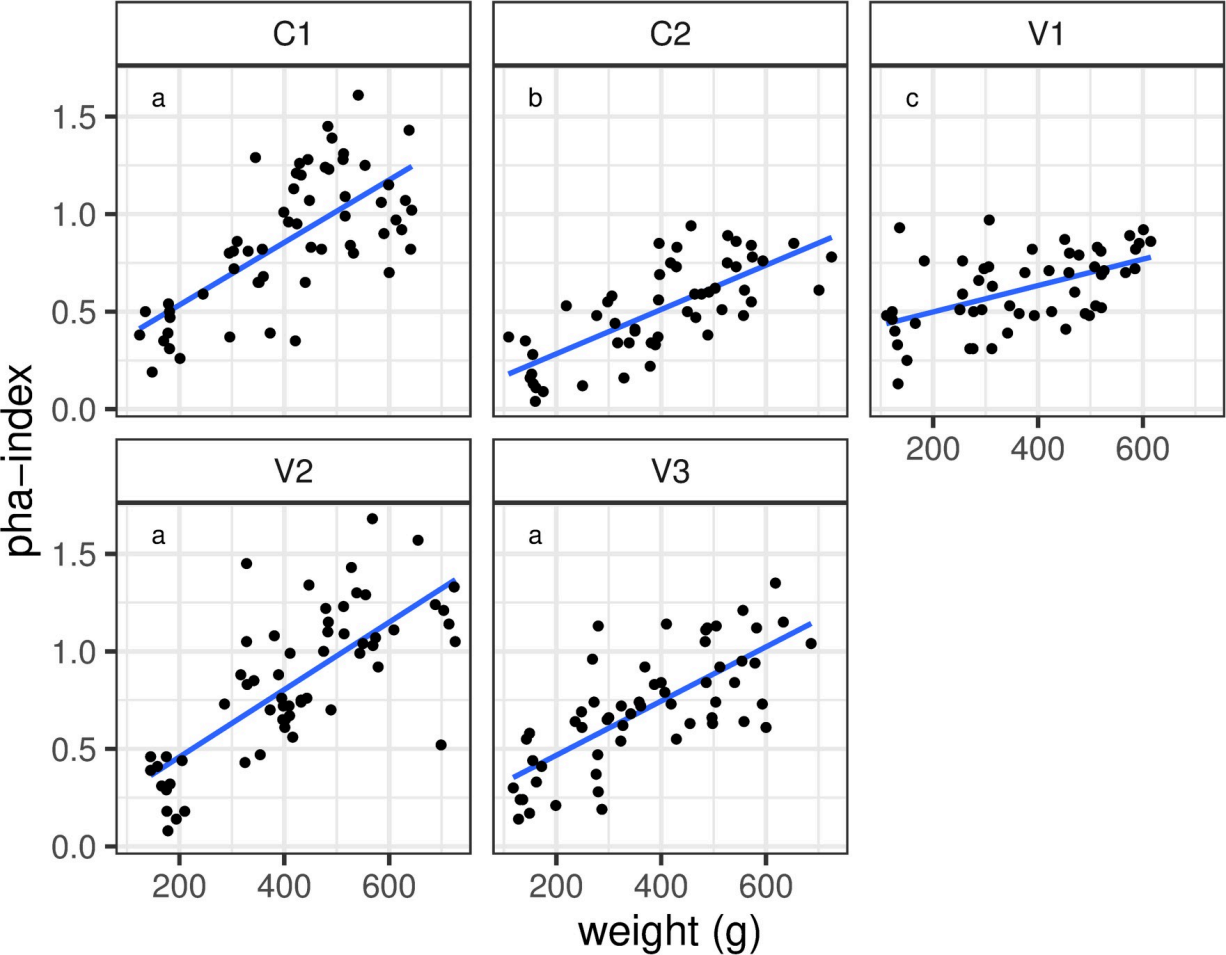
Trend lines presenting the results of the PHA test in correlation with body weight. Small letters indicate significantly different regression slopes between treatments.

In a second model testing the relationship between PHA -index and weight, we see overall increasing PHA -indices with weight from week four onwards (Table 3). While mean values between groups don’t differ significantly, the slope of the relationship is less steep in C2 and V1 compared to C1, while V2 and V3 show no significant difference to C1 (Table 3, Fig 3).

**Table 3:** Coefficients of the generalized least square analysis of the PHA-index dependent on chick weight. The table shows the estimated value, the standard error, the t-value and the significance (p-value) of the terms. The rows represent the groups, the weight and their interactions. Values indicate the differences to the reference value (intercept), which represents pha-index from the control group C1.

	Value	Std.Error	t-value	p-value
(Intercept)	0.184	0.090	2.038	0.043
weight	0.002	0.000	7.778	<0.001
C2	-0.131	0.109	-1.203	0.230
V1	0.182	0.108	1.688	0.093
V2	-0.099	0.124	-0.803	0.423
V3	-0.007	0.112	-0.058	0.954
weight:C2	-0.001	0.000	-2.036	0.043
weight:V1	-0.001	0.000	-3.809	<0.001
weight:V2	0.000	0.000	0.411	0.681
weight:V3	0.000	0.000	-0.912	0.363

### HHA–Test

**Fig 4:**
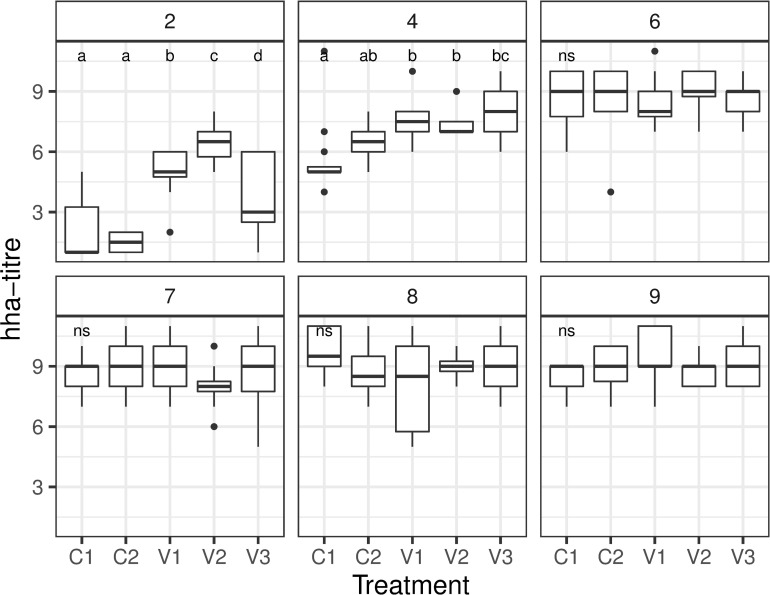
Boxplot presenting the results of the HHA test over the nine weeks of the study. Small letters indicate significant differences between groups within weeks with 2,4,6,7,8,9 indicating the age of the chickens in weeks.

In the beginning, titres of groups V1-V3 were higher than titres of groups C1 and C2 (Fig 4). Weekly testing detected a significant increase of titres per week in all groups without significant differences (Fig 4). Only hemagglutination and no lysis of samples was observed.

### Histopathological examinations

The histological examinations of the immune organs revealed a low to moderate infiltration with granulocytes of the Bursa fabricii across all 5 groups while no alterations were found in the thymus or bone marrow. No differences were detected in gross morphology or histology of Bursa fabricii, thymus and bone marrow between the five groups of chicks.

In the pectoral muscle, mild to moderate degeneration and small isolated inflammatory changes were detected. No differences were found among the control and treatment groups. No gross morphological changes were detected in the liver while the histological examination of the livers revealed occasionally multifocal lymphohistiocytic cell infiltrations and also granulomatous and necrotizing inflammations in most of the investigated chicks. While in the second week the counts of liver inflammation were still low among all groups, numbers increased drastically from the fourth week, and no differences between groups could be detected (Table 4). The degrees of inflammation ranged from mild to mild to moderate and had a granulomatous character, tests for Mycobacteria *spp*. (Ziehl-Neelsen´stain) and fungi (PAS reaction) gave negative results.

**Table 4:** Histopathological examinations for liver, M. pectoralis and skin per week and group (n).

	Group C1						Group V1						Group V2						Group V3						total
**Organ diagnosis / week**	**2**	**4**	**6**	**7**	**8**	**9**	**2**	**4**	**6**	**7**	**8**	**9**	**2**	**4**	**6**	**7**	**8**	**9**	**2**	**4**	**6**	**7**	**8**	**9**	
**Liver inflammations**	1	5	4	5	5	5	5	5	6	5	5	5		3	5	5	5	4	1	4	5	5	5	5	103
**M. pect. changes** *	2	5	3	2	3	5	1	4	2	3	2	3	2	3	3	3	4	3	2	2	4	3	2	2	68
**Skin inflammation**		1	3	2	4	3	1	3	2	4	2	5		4	3	3	5	3	1	3	2	3	4	4	65

*m. pect. changes = degeneration (e. g. hyaline muscle fiber degeneration), and focal myositis of Musculus pectoralis

## Discussion

Previous studies in free-ranging ring-necked pheasants stressed that high chick mortality might form an important factor among the causes for the obviously multifactorial caused decline in population numbers [4, 38–40]. Therefore, we hypothesized that imminent habitat changes according to a human rearrangement of landscape and agriculture as well as the reported drastic decline in insect numbers [41, 42] might have led to an inadequate diet in pheasant chicks.

This study outlines the importance of diet composition in growing ring-necked pheasants. Here, feed intake and body weight development significantly differed between groups, resulting in a maximum feed intake in group V2 which was fed with low energized diet, reflecting in a similar body weight development compared to control groups C1 and C2 in this group (V2) so far and significantly lower body weights in groups V1 and V3. It seems that in group V1, sufficient energy content of the diet has prevented the chicks from a higher feed consumption, leading to a protein-deficiency and retardation of growth, which would be in line with studies on different performances of pheasant chicks according to their diets [16, 43], assuming that energetic malnutrition might be balanced but protein deficiency might not. For control groups C1 and C2, the body weight was comparable to a study on ring-necked pheasants [44]. Throughout the groups, PHA-test results highlighted that a deficient protein diet–simulated in group V1—leads to a lower T-cell mediated immune response rather than a diet that only consisted of low energy (simulated in group V2). There was no significant difference between tests of group V1 and group V3, which was given a low protein as well as a low energy diet. This demonstrates that the lower T-cell mediated response is linked to low protein intake and not to the low energy diet. Results were supported by control groups C1, which was offered an optimal diet, and C2, which was optimally fed but immune suppressed. Although the results of HHA–tests led to no haemolysis in any of the samples, agglutination titres of all experimental groups (V1-V3) were significantly different from control groups, and titres of these groups became significantly higher over time. This is in accordance to Matson et al. [29], who demonstrated that the titres increase with age. The lacking lysis titres might be related to the species examined, as those differ between species. It remains unclear what causes the differences between the control groups and the experimental groups. It can be speculated that especially after at the beginning of the experiment, transport and acquaintance might have caused stress and led to the differences among HHA results that seem to be vague. Although evaluation of immune competence, especially in wild animals, is a task that no laboratory–derived test seems to fully accomplish and has to consider that an immune response is context specific [45], PHA–testing has been used in dietary experiments before [46–48], and the technique is supported by several studies [23, 24, 49, 50]. The outcome of HHA–testing may reflect the difficulties in the applicability of tests in different wildlife species, but at least the hemagglutination titres were measurable in pheasants and titre development act similar as previously described [25] by increasing when the animal ages. This underlines that the methodology is transferrable to pheasants. Although no hemolysis was detected, hemagglutination titres reflect the increase of innate humoral immune competence in all groups during time, compared to a reduced specific T—cell response.

The occurrence of liver inflammations of unknown aetiology (selective pathogen-specific reactions were negative) was similar among groups and therefore an underlying reason should be considered. However, slight inflammations might typically be noticed as an immune response towards environmental pathogens in closed captures. They might also be related to intruding bacteria due to small skin abrasions due to the housing and management of the birds. Here, it is likely that they are not linked specifically to the diet, since all groups were affected. Also, numerous skin abrasions were found in all groups that do not seem to be dietary–specific.

Wildlife research often reveals that multiple factors may lead to observed changes or problems in free-ranging populations, without clearly defining their significance [51, 52]. In this study, the effect of protein availability during the first weeks of pheasant chicks’ life on their immune competence is demonstrated. Starting at the same level with control groups during the first two weeks of life, PHA-tests in growing chicks fed with low-protein diets show significantly lower values over time, facilitating to conclude on the birds actual chances in the field when confronted with their actual habitat. In the literature, even the probability of a correlation of immune competence and predation pressure is discussed [45]. Renaturation of habitats, less pesticide use and a comeback of insects seem to be the first options for a restauration of lowland breeding birds [53–55].

## Conclusions

A reduced T-cell-response in low–protein reared pheasants is likely to lead to a higher susceptibility to diseases and increased mortality in this age class. The confrontation with a variety of pathogens [3] in combination with higher effort to gain appropriate numbers of insects in their diet [14, 56, 57] and consequently diminished immune competence is a valid scenario that might lead to the actual population depletion of free-ranging ring-necked pheasants.

## Supporting information

S1 File (DOCX)

## References

[1] Bauer H-G, Woog F. Nichtheimische Vogelarten (Neozoen) in Deutschland, Teil I: Auftreten, Bestände und Status. Vogelwarte. 2008;46(3):157–194.

[2] Glutz von Blotzheim UN, Bauer KM, Bezzel E. Handbuch der Vögel Mitteleuropas. 2. ed. Wiesbaden: Aula; 1994.

[3] Curland N, Gethoffer F, van Neer A, Ziegler L, Heffels-Redmann U, Lierz M, et al. Investigation into diseases in free-ranging ring-necked pheasants (Phasianus colchicus) in northwestern Germany during population decline with special reference to infectious pathogens. Eur J Wildlife Res. 2018(2):1. doi: 10.1007/s10344-018-1173-2 32214944 PMC7087779

[4] Liebing J, Völker I, Curland N, Wohlsein P, Baumgärtner W, Braune S, et al. Health status of free-ranging ring-necked pheasant chicks (Phasianus colchicus) in North-Western Germany. PLOS ONE. 2020;15(6):e0234044. doi: 10.1371/journal.pone.0234044 32544211 PMC7297342

[5] Strauß E. Wildtiererfassung in Niedersachsen. Niedersächsisches Ministerium f. Ernährung, Landwirtschaft und Verbraucherschutz und Landesentwicklung; 2014. Report No.: 21979839. https://www.ml.niedersachsen.de/startseite/themen/wald_holz_jagd/jagd_in_niedersachsen/jagd-in-niedersachsen-5138.html

[6] Gethöffer F. Entwicklung der Fasanenbesätze. Hannover, Germany: Niedersächsisches Ministerium für Ernährung, Landwirtschaft, Verbraucherschutz und Landesentwicklung; 2011. https://www.ml.niedersachsen.de/startseite/themen/wald_holz_jagd/jagd_in_niedersachsen/jagd-in-niedersachsen-5138.html

[7] Ronnenberg K, Strauss E, Siebert U. Crop diversity loss as primary cause of grey partridge and common pheasant decline in Lower Saxony, Germany. BMC Ecology. 2016; 16(1), 1–15. 27612946 10.1186/s12898-016-0093-9 PMC5016946

[8] Schütte R. EEG stellt Kulturlandschaft auf den Kopf. Landwirtschaftskammer Niedersachsen; 2013 22.11.2013. http://www.lwk-niedersachsen.de/index.cfm/portal/6/nav/355/article/19589.html.

[9] Aebischer NJ. Effects of cropping practices on declining farmland birds during the breeding season. 1997 Brighton Crop Protection Conference—Weeds, Conference Proceedings Vols 1–3. 1997:915–922.

[10] Moreby SJ, Southway SE. Influence of autumn applied herbicides on summer and autumn food available to birds in winter wheat fields in southern England. Agr Ecosyst Environ. 1999;72(3):285–297.

[11] Hawes C, Haughton AJ, Osborne JL, Roy DB, Clark SJ, Perry JN, et al. Responses of plants and invertebrate trophic groups to contrasting herbicide regimes in the Farm Scale Evaluations of genetically modified herbicide-tolerant crops. Philos Trans R Soc Lond B Biol Sci. 2003;358(1439):1899–1913. doi: 10.1098/rstb.2003.1406 14561321 PMC1693274

[12] Heard MS, Hawes C, Champion GT, Clark SJ, Firbank LG, Haughton AJ, et al. Weeds in fields with contrasting conventional and genetically modified herbicide-tolerant crops. I. Effects on abundance and diversity. Philos Trans R Soc Lond B Biol Sci. 2003;358(1439):1819–1832. doi: 10.1098/rstb.2003.1402 14561316 PMC1693279

[13] Wittmann P. Der Edelfasan (Phasianus colchicus) seine Naturgeschichte, Aufzucht und Hege, Jagd und Benutzung. Wien: k. u. k. Hofbuchhändler; 1891. 245 p.

[14] Hill DA, Robertson P. The pheasant: ecology, management and conservation. Mead O, editor, Oxford, Uk: Blackwell Scientific Publication; 1988. 281 p.

[15] Đorđević N, Popović Z, Beuković М, Beuković D, Đorđević M, editors. The importance of protein quantity and quality for different pheasant categorise in aviaries and nature. International symposium on hunting „Modern aspects of sustainable management of game population”, Zemun-Belgrade, Serbia; 2012.

[16] Djordjević M, Pekeč S, Popović Z, Djordjević N. Influence of dietary protein levels on production results and mortality in pheasants reared under controlled conditions. Acta veterinaria. 2010;60(1):79–88.

[17] Gonzalez G, Sorci G, Moller AP, Ninni P, Haussy C, De Lope F. Immunocompetence and condition-dependent sexual advertisement in male house sparrows (Passer domesticus). J Anim Ecol. 1999;68(6):1225–1234.

[18] Tella JL, Forero MG, Bertellotti M, Donazar JA, Blanco G, Ceballos O. Offspring body condition and immunocompetence are negatively affected by high breeding densities in a colonial seabird: a multiscale approach. P Roy Soc B-Biol Sci. 2001;268(1475):1455–1461. 10.1098/rspb.2001.1688 PMC1088763 11454288

[19] Birkhead TR, Fletcher F, Pellatt EJ. Nestling diet, secondary sexual traits and fitness in the zebra finch. Proceedings of the Royal Society of London Series B: Biological Sciences. 1999;266(1417):385–390.

[20] Saino N, Calza S, pape Moller A. Immunocompetence of nestling barn swallows in relation to brood size and parental effort. J Anim Ecol. 1997:827–836.

[21] Potts GR. Recent Changes in Farmland Fauna with Special Reference to Decline of Grey Partridge. Bird Study. 1970;17(2):145–166.

[22] Fairbrother A, Smits J, Grasman KA. Avian immunotoxicology. J Toxicol Env Heal B. 2004;7(2):105–137. doi: 10.1080/10937400490258873 14769546

[23] Santiago-Quesada F, Albano N, Castillo-Guerrero JA, Fernandez G, Gonzalez-Medina E, Sanchez-Guzman JM, et al. Secondary phytohaemagglutinin (PHA) swelling response is a good indicator of T-cell-mediated immunity in free-living birds. Ibis. 2015(4):767.

[24] Tella JL, Lemus JA, Carrete M, Blanco G. The PHA Test Reflects Acquired T-Cell Mediated Immunocompetence in Birds. Plos One. 2008;3(9): e3295. doi: 10.1371/journal.pone.0003295 18820730 PMC2546448

[25] Gonzalez G, Sorci G, de Lope F. Seasonal variation in the relationship between cellular immune response and badge size in male house sparrows (Passer domesticus). Behav Ecol Sociobiol. 1999;46(2):117–122.

[26] Smits JE, Bortolotti GR, Tella JL. Simplifying the phytohaemagglutinin skin-testing technique in studies of avian immunocompetence. Funct Ecol. 1999;13(4):567–572.

[27] Tella JL, Bortolotti GR, Forero MG, Dawson RD. Environmental and genetic variation in T-cell-mediated immune response of fledgling American kestrels. Oecologia. 2000;123(4):453–459. doi: 10.1007/s004420000331 28308752

[28] Salaberria C, Muriel J, de Luna M, Gil D, Puerta M. The PHA test as an indicator of phagocytic activity in a passerine bird. Plos One. 2013;8(12):e84108. doi: 10.1371/journal.pone.0084108 24391896 PMC3877195

[29] Matson KD, Ricklefs RE, Klasing KC. A hemolysis-hemagglutination assay for characterizing constitutive innate humoral immunity in wild and domestic birds. Dev Comp Immunol. 2005;29(3):275–286. doi: 10.1016/j.dci.2004.07.006 15572075

[30] Anonymous. Leitfaden Landwirtschaft Geflügelmast. 2021; QS Fachgesellschaft Geflügel GmbH; www.q-s.de.

[31] Golze DM, Wehlitz R. Fachartikel Fasanenhaltung. Kuratorium für Technik und Bauwesen in der Landwirtschaft e V (KTBL). 2014. https://www.ktbl.de/themen/fasanenhaltung

[32] Gauly M. Agricultural pheasant rearing / Landwirtschaftliche Fasanenhaltung. Stuttgart (Hohenheim): E. Ulmer; 1994. 108 p.

[33] C National Research Council BoA, Subcommittee on Poultry Nutrition. National Research Council Nutrient Requirements of Poultry—Ninth Revised Edition (1994): National Academies Press; 1994.

[34] Smits JE, Bortolotti GR, Tella JL. Measurement repeatability and the use of controls in PHA assays: Reply. Funct Ecol. 2001;15(6):814–817.

[35] Wegmann TG, Smithies O. A Simple Hemagglutination System Requiring Small Amounts of Red Cells and Antibodies. Transfusion. 1966;6(1):67.

[36] Bhullar Kaur R, Bhullar A, Vanaki S, Puranik RS, Sudhakara M, Kamat M. A comparative histopathological & bacteriological insight into periapical lesions: An analysis of 62 lesions from north Karnataka. Indian Journal of Dentistry. 2013;4(4):200–206.

[37] Fischer D, Lierz M. Diagnostic Procedures and Available Techniques for the Diagnosis of Aspergillosis in Birds. Journal of Exotic Pet Medicine. 2015;24(3):283–295.

[38] Fischer L, Liebing J, Völker I, Baudler L, Gethöffer F, Voigt U, et al. Occurrence and relevance of Mycoplasma spp. in free-ranging pheasants from northwestern Germany. European Journal of Wildlife Research. 2022;68(1):7.

[39] Curland N, Gethöffer F, van Neer A, Ziegler L, Heffels-Redmann U, Lierz M, et al. Investigation into diseases in free-ranging ring-necked pheasants (Phasianus colchicus) in northwestern Germany during population decline with special reference to infectious pathogens. European journal of wildlife research. 2018;64(2):1–15. doi: 10.1007/s10344-018-1173-2 32214944 PMC7087779

[40] Gethöffer F, Curland N, Voigt U, Woelfing B, Ludwig T, Heffels-Redmann U, et al. Seroprevalences of specific antibodies against avian pathogens in free-ranging ring-necked pheasants (Phasianus colchicus) in Northwestern Germany. PLOS ONE. 2021;16(8):e0255434. doi: 10.1371/journal.pone.0255434 34347834 PMC8336876

[41] Goulson D. The insect apocalypse, and why it matters. Current Biology. 2019;29(19):R967–R971. doi: 10.1016/j.cub.2019.06.069 31593678

[42] Leather SR. “Ecological Armageddon”-more evidence for the drastic decline in insect numbers. Annals of Applied Biology. 2017;172(1):1–3.

[43] Delfelder CJ. Effects of nutrient levels on pen-raised ring-necked pheasant performance and feather growth parameters 2021. Kansas State University; 51 pp.

[44] Kuźniacka J, Adamski M. Growth rate of body weight and measurements in pheasants reared up to the 24th week of life (Short Communication). Archiv fur Tierzucht. 2010;53: 360–367

[45] Viney ME, Riley EM, Buchanan KL. Optimal immune responses: immunocompetence revisited. Trends Ecol Evol. 2005;20(12):665–669. doi: 10.1016/j.tree.2005.10.003 16701455

[46] Lessard M, Hutchings D, Cave NA. Cell-mediated and humoral immune responses in broiler chickens maintained on diets containing different levels of vitamin A. Poult Sci. 1997;76(10):1368–1378. doi: 10.1093/ps/76.10.1368 9316112

[47] Lochmiller RL, Vestey MR, Boren JC. Relationship between Protein Nutritional-Status and Immunocompetence in Northern Bobwhite Chicks. Auk. 1993;110(3):503–510.

[48] Alonso-Alvarez C, Tella JL. Effects of experimental food restriction and body-mass changes on the avian T-cell-mediated immune response. Can J Zool. 2001;79(1):101–105.

[49] Martin LB, Han P, Lewittes J, Kuhlman JR, Klasing KC, Wikelski M. Phytohemagglutinin-Induced Skin Swelling in Birds: Histological Support for a Classic Immunoecological Technique. 2006:290.

[50] Goto N, Kodama H, Okada K, Fujimoto Y. Suppression of phytohemagglutinin skin response in thymectomized chickens. Poult Sci. 1978;57(1):246–250. doi: 10.3382/ps.0570246 674011

[51] Hallmann CA, Foppen RP, van Turnhout CA, de Kroon H, Jongejans E. Declines in insectivorous birds are associated with high neonicotinoid concentrations. Nature. 2014;511(7509):341–343. doi: 10.1038/nature13531 25030173

[52] Tillmann JE, Ronnenberg K. Assessment of habitat-specific food availability using human imprinted Grey Partridge (Perdix perdix) chicks. Ornis Fennica. 2015;92(2):87–100.

[53] Henderson IG, Ravenscroft N, Smith G, Holloway S. Effects of crop diversification and low pesticide inputs on bird populations on arable land. Agr Ecosyst Environ. 2009;129(1–3):149–156.

[54] Fuller RJ, editor Responses of birds to organic arable farming: Mechanisms and evidence. 1997 Brighton Crop Protection Conference—Weeds, Conference Proceedings Vols 1–3; 1997.

[55] Sotherton NW. Land use changes and the decline of farmland wildlife: An appraisal of the set-aside approach. Biol Conserv. 1998;83(3):259–268.

[56] Hill DA. The Feeding Ecology and Survival of Pheasant Chicks on Arable Farmland. J Appl Ecol. 1985;22(3):645–654.

[57] Doxon ED, Carroll JP. Feeding Ecology of Ring-Necked Pheasant and Northern Bobwhite Chicks in Conservation Reserve Program Fields. J Wildl Manage. 2010;74(2):249–256.

