# Supplementary material for: The modulating effect of food composition on the immune system in growing ring-necked pheasants (Phasianus colchicus)
Source: PLoS One. 2022 Nov 7;17(11):e0277236. doi: 10.1371/journal.pone.0277236 (PMC9639844; doi:10.1371/journal.pone.0277236)
Supplement: S1 File — (DOCX) [file pone.0277236.s001.docx]

**The modulating effect of food composition on the immune system in growing ring-necked pheasants (***Phasianus colchicus***)**

Friederike Gethöffer^1*^, Jennifer Liebing^1^, Katrin Ronnenberg^5,^ Nele Curland^1,^ Christina Puff², Peter Wohlsein², Wolfgang Baumgärtner², Bianca Bücking^3^, Ursula Heffels-Redmann^3^, Ulrich Voigt^1^, Christian Sonne^4^, Michael Lierz^3^, Ursula Siebert^1,4^

**Supporting Information S1**

S1 Table. Composition and analytical components of the feed of the two control (C1-C2) and three experimental groups (V1-V3).

|  | Control (C1/C2) | V1 | V2 | V3 |
| --- | --- | --- | --- | --- |
| composition in % |  |  |  |  |
| fat (soybean oil) | 2.30 | 2.50 | 2.00 | 2.00 |
| wheat | 29.40 | 33.20 | 35.74 | 50.75 |
| wheat bran | 0.00 | 0.00 | 10.00 | 10.00 |
| corn | 30.00 | 39.12 | 2.00 | 2.00 |
| lucerne meal |  |  | 3.00 | 3.00 |
| oat grains | 0.0 | 0.0 | 5.0 | 5.0 |
|  |  |  |  |  |
| CaCO_3_ | 1.20 | 1.200 | 1.500 | 1.300 |
| NaCl | 0.10 | 0.100 | 0.100 | 0.100 |
| Dicalc. Phosphate | 0.80 | 1.000 | 0.500 | 0.800 |
| methionine | 0.11 | 0.030 | 0.110 | 0.050 |
| lysine | 0.100 | 0.050 | 0.050 | 0.100 |
| G13 vit | 2.800 | 2.800 | 2.800 | 2.800 |
| fibre cell | 0.000 | 0.000 | 2.000 | 2.000 |
| bolus alba |  |  | 2.000 | 2.000 |
| analytical components (g/kg) |  |  |  |  |
| raw protein | 269.9 | 197.3 | 276.2 | 201.2 |
| raw fat | 44.7 | 48.6 | 40.4 | 41.0 |
| raw fibre | 29.8 | 28.7 | 58.1 | 56.8 |
| sugar | 48.2 | 37.6 | 51.5 | 39.0 |
| amylum | 367 | 441 | 258 | 336 |
| metabolic energy | 12.5 | 12.6 | 10.6 | 10.6 |
| calcium | 13.26 | 13.44 | 13.96 | 13.65 |
| phosphor | 8.75 | 7.84 | 9.19 | 8.33 |
| natrium | 1.51 | 1.52 | 1.58 | 1.59 |
| potassium | 8.34 | 6.21 | 7.74 | 5.35 |
| chlorid | 1.06 | 1.08 | 1.22 | 1.26 |
| methionin | 6.07 | 4.40 | 6.09 | 4.54 |
| cystein | 5.52 | 3.61 | 5.49 | 3.75 |
| lysine | 15.6 | 10.8 | 15.8 | 11.1 |
| tryptophane | 2.86 | 1.91 | 2.89 | 1.84 |
| threonine | 11.69 | 7.96 | 11.26 | 7.90 |
| methionine + cystine | 11.59 | 8.01 | 11.59 | 8.29 |

S 1 Table. Coefficients of the generalized least square analyses of chick weight dependent on group and age in weeks. The table shows the estimated value, the standard error, the t-value and the significance (p-value) of the terms. The rows represent the groups, the age and their interactions. Values indicate the differences to the reference value (intercept), which represents chicks weight from the control group C1 at the age of two weeks.

|  |  |  |  |  |
| --- | --- | --- | --- | --- |
|  | Value | Std.Error | t-value | p-value |
| (Intercept) | 58.250 | 1.795 | 32.443 | <.0001 |
| week 4 | 109.650 | 6.772 | 16.191 | <.0001 |
| week 6 | 263.750 | 12.160 | 21.690 | <.0001 |
| week 7 | 387.667 | 15.808 | 24.524 | <.0001 |
| week 8 | 461.917 | 19.871 | 23.245 | <.0001 |
| week 9 | 498.833 | 24.332 | 20.501 | <.0001 |
| C2 | -1.417 | 2.539 | -0.558 | 0.577 |
| V1 | -2.750 | 2.539 | -1.083 | 0.280 |
| V2 | 7.500 | 2.539 | 2.954 | 0.003 |
| V3 | -2.000 | 2.539 | -0.788 | 0.432 |
| week 4: C2 | -15.372 | 9.822 | -1.565 | 0.119 |
| week 6: C2 | -12.083 | 18.019 | -0.671 | 0.503 |
| week 7: C2 | -39.682 | 22.852 | -1.736 | 0.084 |
| week 8: C2 | -34.333 | 28.102 | -1.222 | 0.223 |
| week 9: C2 | 40.633 | 36.082 | 1.126 | 0.261 |
| week 4: V1 | -26.950 | 9.577 | -2.814 | 0.005 |
| week 6: V1 | -29.432 | 17.575 | -1.675 | 0.095 |
| week 7: V1 | -71.076 | 22.852 | -3.110 | 0.002 |
| week 8: V1 | -1.017 | 29.463 | -0.035 | 0.973 |
| week 9: V1 | -25.970 | 35.181 | -0.738 | 0.461 |
| week 4: V2 | 0.350 | 9.199 | 0.038 | 0.970 |
| week 6: V2 | 28.417 | 17.197 | 1.652 | 0.100 |
| week 7: V2 | -28.000 | 22.356 | -1.252 | 0.211 |
| week 8: V2 | 20.750 | 28.102 | 0.738 | 0.461 |
| week 9: V2 | 29.167 | 34.411 | 0.848 | 0.397 |
| week 4: V3 | -16.627 | 9.373 | -1.774 | 0.077 |
| week 6: V3 | -39.636 | 17.575 | -2.255 | 0.025 |
| week 7: V3 | -79.667 | 22.356 | -3.564 | <.0001 |
| week 8: V3 | -32.712 | 28.729 | -1.139 | 0.256 |
| week 9: V3 | 7.167 | 34.411 | 0.208 | 0.835 |

S1 Table. Minimal data set for PHA – index evaluation.

| group | week | pha_index |
| --- | --- | --- |
| K2 | 2 | 0,30 |
| K2 | 2 | 0,79 |
| K2 | 2 | 0,60 |
| K2 | 2 | 0,42 |
| K2 | 2 | 0,82 |
| K2 | 2 | 0,63 |
| K2 | 2 | 0,17 |
| K2 | 2 | 0,10 |
| K2 | 2 | 0,72 |
| K2 | 2 | 1,15 |
| K2 | 2 | 0,81 |
| K2 | 2 | 0,86 |
| K1 | 2 | 1,33 |
| K1 | 2 | 1,00 |
| K1 | 2 | 1,23 |
| K1 | 2 | 0,97 |
| K1 | 2 | 0,34 |
| K1 | 2 | 0,35 |
| K1 | 2 | 1,11 |
| K1 | 2 | 0,74 |
| K1 | 2 | 0,47 |
| K1 | 2 | 0,70 |
| K1 | 2 | 0,57 |
| K1 | 2 | 0,54 |
| V1 | 2 | 0,87 |
| V1 | 2 | 0,38 |
| V1 | 2 | 1,67 |
| V1 | 2 | 0,16 |
| V1 | 2 | 0,82 |
| V1 | 2 | 0,63 |
| V1 | 2 | 0,20 |
| V1 | 2 | 0,19 |
| V1 | 2 | 1,43 |
| V1 | 2 | 1,19 |
| V1 | 2 | 0,85 |
| V1 | 2 | 1,11 |
| V2 | 2 | 0,46 |
| V2 | 2 | 1,02 |
| V2 | 2 | 0,50 |
| V2 | 2 | 0,80 |
| V2 | 2 | 0,84 |
| V2 | 2 | 0,63 |
| V2 | 2 | 1,39 |
| V2 | 2 | 0,61 |
| V2 | 2 | 1,41 |
| V2 | 2 | 0,83 |
| V2 | 2 | 0,71 |
| V2 | 2 | 0,79 |
| V3 | 2 | 0,92 |
| V3 | 2 | 0,29 |
| V3 | 2 | 0,55 |
| V3 | 2 | 0,66 |
| V3 | 2 | 0,45 |
| V3 | 2 | 0,46 |
| V3 | 2 | 1,46 |
| V3 | 2 | 0,33 |
| V3 | 2 | 0,23 |
| V3 | 2 | 0,58 |
| V3 | 2 | 0,85 |
| V3 | 2 | 0,83 |
| K1 | 4 | 0,54 |
| K1 | 4 | 0,38 |
| K1 | 4 | 0,47 |
| K1 | 4 | 0,19 |
| K1 | 4 | 0,31 |
| K1 | 4 | 0,50 |
| K1 | 4 | 0,50 |
| K1 | 4 | 0,26 |
| K1 | 4 | 0,39 |
| K1 | 4 | 0,35 |
| K2 | 4 | 0,11 |
| K2 | 4 | 0,13 |
| K2 | 4 | 0,18 |
| K2 | 4 | 0,37 |
| K2 | 4 | 0,04 |
| K2 | 4 | 0,16 |
| K2 | 4 | 0,35 |
| K2 | 4 | 0,09 |
| K2 | 4 | 0,28 |
| V1 | 4 | 0,13 |
| V1 | 4 | 0,40 |
| V1 | 4 | 0,48 |
| V1 | 4 | 0,50 |
| V1 | 4 | 0,44 |
| V1 | 4 | 0,46 |
| V1 | 4 | 0,93 |
| V1 | 4 | 0,33 |
| V1 | 4 | 0,76 |
| V1 | 4 | 0,25 |
| V2 | 4 | 0,44 |
| V2 | 4 | 0,46 |
| V2 | 4 | 0,46 |
| V2 | 4 | 0,14 |
| V2 | 4 | 0,31 |
| V2 | 4 | 0,18 |
| V2 | 4 | 0,08 |
| V2 | 4 | 0,32 |
| V2 | 4 | 0,39 |
| V2 | 4 | 0,18 |
| V2 | 4 | 0,29 |
| V2 | 4 | 0,41 |
| V3 | 4 | 0,21 |
| V3 | 4 | 0,17 |
| V3 | 4 | 0,58 |
| V3 | 4 | 0,14 |
| V3 | 4 | 0,55 |
| V3 | 4 | 0,41 |
| V3 | 4 | 0,24 |
| V3 | 4 | 0,33 |
| V3 | 4 | 0,24 |
| V3 | 4 | 0,44 |
| V3 | 4 | 0,30 |
| K1 | 6 | 0,86 |
| K1 | 6 | 0,80 |
| K1 | 6 | 0,81 |
| K1 | 6 | 1,29 |
| K1 | 6 | 0,81 |
| K1 | 6 | 0,65 |
| K1 | 6 | 0,65 |
| K1 | 6 | 0,59 |
| K1 | 6 | 0,39 |
| K1 | 6 | 0,72 |
| K1 | 6 | 0,37 |
| K1 | 6 | 0,68 |
| K2 | 6 | 0,40 |
| K2 | 6 | 0,16 |
| K2 | 6 | 0,48 |
| K2 | 6 | 0,55 |
| K2 | 6 | 0,34 |
| K2 | 6 | 0,34 |
| K2 | 6 | 0,37 |
| K2 | 6 | 0,12 |
| K2 | 6 | 0,53 |
| K2 | 6 | 0,44 |
| V1 | 6 | 0,39 |
| V1 | 6 | 0,66 |
| V1 | 6 | 0,31 |
| V1 | 6 | 0,73 |
| V1 | 6 | 0,76 |
| V1 | 6 | 0,63 |
| V1 | 6 | 0,50 |
| V1 | 6 | 0,51 |
| V1 | 6 | 0,31 |
| V1 | 6 | 0,59 |
| V1 | 6 | 0,31 |
| V2 | 6 | 0,85 |
| V2 | 6 | 0,74 |
| V2 | 6 | 0,73 |
| V2 | 6 | 0,67 |
| V2 | 6 | 0,88 |
| V2 | 6 | 0,47 |
| V2 | 6 | 0,61 |
| V2 | 6 | 0,72 |
| V2 | 6 | 0,70 |
| V2 | 6 | 0,43 |
| V2 | 6 | 0,83 |
| V2 | 6 | 1,05 |
| V3 | 6 | 0,37 |
| V3 | 6 | 0,69 |
| V3 | 6 | 0,61 |
| V3 | 6 | 0,28 |
| V3 | 6 | 0,19 |
| V3 | 6 | 0,47 |
| V3 | 6 | 0,62 |
| V3 | 6 | 0,74 |
| V3 | 6 | 0,65 |
| V3 | 6 | 0,66 |
| V3 | 6 | 0,96 |
| K1 | 7 | 1,21 |
| K1 | 7 | 1,07 |
| K1 | 7 | 1,45 |
| K1 | 7 | 0,83 |
| K1 | 7 | 0,99 |
| K1 | 7 | 1,28 |
| K1 | 7 | 0,65 |
| K1 | 7 | 1,13 |
| K1 | 7 | 0,35 |
| K1 | 7 | 0,82 |
| K1 | 7 | 1,09 |
| K1 | 7 | 1,20 |
| K2 | 7 | 0,34 |
| K2 | 7 | 0,73 |
| K2 | 7 | 0,69 |
| K2 | 7 | 0,61 |
| K2 | 7 | 0,50 |
| K2 | 7 | 0,56 |
| K2 | 7 | 0,33 |
| K2 | 7 | 0,22 |
| K2 | 7 | 0,41 |
| K2 | 7 | 0,58 |
| K2 | 7 | 0,75 |
| V1 | 7 | 0,80 |
| V1 | 7 | 0,71 |
| V1 | 7 | 0,53 |
| V1 | 7 | 0,41 |
| V1 | 7 | 0,50 |
| V1 | 7 | 0,49 |
| V1 | 7 | 0,70 |
| V1 | 7 | 0,48 |
| V1 | 7 | 0,51 |
| V1 | 7 | 0,72 |
| V1 | 7 | 0,97 |
| V2 | 7 | 0,70 |
| V2 | 7 | 0,75 |
| V2 | 7 | 0,65 |
| V2 | 7 | 1,00 |
| V2 | 7 | 0,72 |
| V2 | 7 | 0,76 |
| V2 | 7 | 0,99 |
| V2 | 7 | 0,88 |
| V2 | 7 | 0,76 |
| V2 | 7 | 0,65 |
| V2 | 7 | 1,34 |
| V2 | 7 | 0,56 |
| V3 | 7 | 0,74 |
| V3 | 7 | 0,54 |
| V3 | 7 | 0,84 |
| V3 | 7 | 1,13 |
| V3 | 7 | 0,55 |
| V3 | 7 | 0,92 |
| V3 | 7 | 0,83 |
| V3 | 7 | 0,72 |
| V3 | 7 | 0,72 |
| V3 | 7 | 0,64 |
| V3 | 7 | 0,66 |
| V3 | 7 | 0,79 |
| K1 | 8 | 1,43 |
| K1 | 8 | 1,28 |
| K1 | 8 | 1,15 |
| K1 | 8 | 0,80 |
| K1 | 8 | 1,26 |
| K1 | 8 | 0,90 |
| K1 | 8 | 0,82 |
| K1 | 8 | 0,70 |
| K1 | 8 | 0,96 |
| K1 | 8 | 0,95 |
| K1 | 8 | 1,23 |
| K1 | 8 | 1,25 |
| K2 | 8 | 0,94 |
| K2 | 8 | 0,60 |
| K2 | 8 | 0,47 |
| K2 | 8 | 0,85 |
| K2 | 8 | 0,51 |
| K2 | 8 | 0,59 |
| K2 | 8 | 0,59 |
| K2 | 8 | 0,38 |
| K2 | 8 | 0,83 |
| K2 | 8 | 0,48 |
| K2 | 8 | 0,73 |
| K2 | 8 | 0,89 |
| V1 | 8 | 0,69 |
| V1 | 8 | 0,70 |
| V1 | 8 | 0,52 |
| V1 | 8 | 0,89 |
| V1 | 8 | 0,82 |
| V1 | 8 | 0,82 |
| V1 | 8 | 0,87 |
| V1 | 8 | 0,72 |
| V1 | 8 | 0,53 |
| V1 | 8 | 0,70 |
| V2 | 8 | 1,33 |
| V2 | 8 | 1,22 |
| V2 | 8 | 1,30 |
| V2 | 8 | 0,99 |
| V2 | 8 | 1,03 |
| V2 | 8 | 1,57 |
| V2 | 8 | 1,10 |
| V2 | 8 | 1,43 |
| V2 | 8 | 1,15 |
| V2 | 8 | 1,23 |
| V2 | 8 | 1,09 |
| V2 | 8 | 1,04 |
| V3 | 8 | 0,63 |
| V3 | 8 | 1,14 |
| V3 | 8 | 0,73 |
| V3 | 8 | 0,84 |
| V3 | 8 | 1,21 |
| V3 | 8 | 0,68 |
| V3 | 8 | 0,63 |
| V3 | 8 | 0,84 |
| V3 | 8 | 0,74 |
| V3 | 8 | 1,35 |
| V3 | 8 | 0,92 |
| K1 | 9 | 1,39 |
| K1 | 9 | 1,07 |
| K1 | 9 | 0,82 |
| K1 | 9 | 1,61 |
| K1 | 9 | 0,97 |
| K1 | 9 | 0,92 |
| K1 | 9 | 1,31 |
| K1 | 9 | 1,02 |
| K1 | 9 | 0,84 |
| K1 | 9 | 1,24 |
| K1 | 9 | 1,06 |
| K1 | 9 | 1,01 |
| K2 | 9 | 0,84 |
| K2 | 9 | 0,75 |
| K2 | 9 | 0,62 |
| K2 | 9 | 0,55 |
| K2 | 9 | 0,78 |
| K2 | 9 | 0,61 |
| K2 | 9 | 0,76 |
| K2 | 9 | 0,78 |
| K2 | 9 | 0,85 |
| K2 | 9 | 0,86 |
| V1 | 9 | 0,73 |
| V1 | 9 | 0,60 |
| V1 | 9 | 0,49 |
| V1 | 9 | 0,85 |
| V1 | 9 | 0,81 |
| V1 | 9 | 0,48 |
| V1 | 9 | 0,79 |
| V1 | 9 | 0,92 |
| V1 | 9 | 0,71 |
| V1 | 9 | 0,83 |
| V1 | 9 | 0,86 |
| V2 | 9 | 1,68 |
| V2 | 9 | 1,07 |
| V2 | 9 | 1,05 |
| V2 | 9 | 1,24 |
| V2 | 9 | 1,29 |
| V2 | 9 | 1,11 |
| V2 | 9 | 0,52 |
| V2 | 9 | 0,92 |
| V2 | 9 | 1,08 |
| V2 | 9 | 1,45 |
| V2 | 9 | 1,14 |
| V2 | 9 | 1,21 |
| V3 | 9 | 1,15 |
| V3 | 9 | 1,12 |
| V3 | 9 | 1,04 |
| V3 | 9 | 0,95 |
| V3 | 9 | 1,05 |
| V3 | 9 | 0,61 |
| V3 | 9 | 0,73 |
| V3 | 9 | 1,11 |
| V3 | 9 | 0,64 |
| V3 | 9 | 0,94 |
| V3 | 9 | 1,13 |
| V3 | 9 | 1,12 |

Table S4: Minimal data set for HHA – evaluation:

| group | week | titer |
| --- | --- | --- |
| K2 | 2 | 1 |
| K2 | 2 | 2 |
| K2 | 2 | 1 |
| K2 | 2 | 1 |
| K2 | 2 | 2 |
| K2 | 2 | 2 |
| K2 | 2 | 2 |
| K2 | 2 | 2 |
| K2 | 2 | 2 |
| K2 | 2 | <1 |
| K2 | 2 | <1 |
| K2 | 2 | <1 |
| K1 | 2 | 5 |
| K1 | 2 | 4 |
| K1 | 2 | <1 |
| K1 | 2 | <1 |
| K1 | 2 | 1 |
| K1 | 2 | 1 |
| K1 | 2 | 3 |
| K1 | 2 | 1 |
| K1 | 2 | <1 |
| K1 | 2 | 5 |
| K1 | 2 | 1 |
| K1 | 2 | 1 |
| V1 | 2 | 2 |
| V1 | 2 | 4 |
| V1 | 2 | 4 |
| V1 | 2 | 6 |
| V1 | 2 | 5 |
| V1 | 2 | 5 |
| V1 | 2 | 5 |
| V1 | 2 | 5 |
| V1 | 2 | 5 |
| V1 | 2 | 6 |
| V1 | 2 | 6 |
| V1 | 2 | 6 |
| V2 | 2 | 6 |
| V2 | 2 | 5 |
| V2 | 2 | 5 |
| V2 | 2 | 5 |
| V2 | 2 | 7 |
| V2 | 2 | 8 |
| V2 | 2 | 7 |
| V2 | 2 | 6 |
| V2 | 2 | 7 |
| V2 | 2 | 6 |
| V2 | 2 | 7 |
| V2 | 2 | 7 |
| V3 | 2 | 3 |
| V3 | 2 | 6 |
| V3 | 2 | 6 |
| V3 | 2 | 3 |
| V3 | 2 | 3 |
| V3 | 2 | 6 |
| V3 | 2 | 3 |
| V3 | 2 | 5 |
| V3 | 2 | <1 |
| V3 | 2 | <1 |
| V3 | 2 | 6 |
| V3 | 2 | <1 |
| K1 | 4 | 5 |
| K1 | 4 | 5 |
| K1 | 4 | 5 |
| K1 | 4 | 4 |
| K1 | 4 | 5 |
| K1 | 4 | 7 |
| K1 | 4 | 11 |
| K1 | 4 | 5 |
| K1 | 4 | 6 |
| K1 | 4 | 5 |
| K1 | 4 | 5 |
| K1 | 4 | 5 |
| K2 | 4 | 6 |
| K2 | 4 | 5 |
| K2 | 4 | 6 |
| K2 | 4 | 6 |
| K2 | 4 | 6 |
| K2 | 4 | 6 |
| K2 | 4 | 7 |
| K2 | 4 | 7 |
| K2 | 4 | 8 |
| K2 | 4 | 7 |
| K2 | 4 | 7 |
| K2 | 4 | 7 |
| V1 | 4 | 7 |
| V1 | 4 | 7 |
| V1 | 4 | 7 |
| V1 | 4 | 7 |
| V1 | 4 | 7 |
| V1 | 4 | 10 |
| V1 | 4 | 8 |
| V1 | 4 | 8 |
| V1 | 4 | 8 |
| V1 | 4 | 8 |
| V1 | 4 | 8 |
| V1 | 4 | 6 |
| V2 | 4 | 7 |
| V2 | 4 | 9 |
| V2 | 4 | 7 |
| V2 | 4 | 7 |
| V2 | 4 | 7 |
| V2 | 4 | 7 |
| V2 | 4 | 7 |
| V2 | 4 | 9 |
| V2 | 4 | 7 |
| V2 | 4 | 9 |
| V2 | 4 | 7 |
| V2 | 4 | 7 |
| V3 | 4 | 7 |
| V3 | 4 | 9 |
| V3 | 4 | 8 |
| V3 | 4 | 6 |
| V3 | 4 | 7 |
| V3 | 4 | 9 |
| V3 | 4 | 7 |
| V3 | 4 | 9 |
| V3 | 4 | 8 |
| V3 | 4 | 9 |
| V3 | 4 | 8 |
| V3 | 4 | 10 |
| K1 | 6 | 10 |
| K1 | 6 | 7 |
| K1 | 6 | 8 |
| K1 | 6 | 10 |
| K1 | 6 | 9 |
| K1 | 6 | 9 |
| K1 | 6 | 8 |
| K1 | 6 | 10 |
| K1 | 6 | 10 |
| K1 | 6 | 6 |
| K1 | 6 | 10 |
| K1 | 6 | 7 |
| K2 | 6 | 10 |
| K2 | 6 | 10 |
| K2 | 6 | 4 |
| K2 | 6 | 4 |
| K2 | 6 | 8 |
| K2 | 6 | 8 |
| K2 | 6 | 10 |
| K2 | 6 | 9 |
| K2 | 6 | 9 |
| K2 | 6 | 8 |
| K2 | 6 | 10 |
| K2 | 6 | 9 |
| V1 | 6 | 8 |
| V1 | 6 | 8 |
| V1 | 6 | 7 |
| V1 | 6 | 7 |
| V1 | 6 | 9 |
| V1 | 6 | 11 |
| V1 | 6 | 8 |
| V1 | 6 | 10 |
| V1 | 6 | 9 |
| V1 | 6 | 9 |
| V1 | 6 | 7 |
| V1 | 6 | 8 |
| V2 | 6 | 10 |
| V2 | 6 | 9 |
| V2 | 6 | 8 |
| V2 | 6 | 8 |
| V2 | 6 | 9 |
| V2 | 6 | 7 |
| V2 | 6 | 9 |
| V2 | 6 | 10 |
| V2 | 6 | 10 |
| V2 | 6 | 9 |
| V2 | 6 | 9 |
| V2 | 6 | 10 |
| V3 | 6 | 9 |
| V3 | 6 | 8 |
| V3 | 6 | 9 |
| V3 | 6 | 8 |
| V3 | 6 | 8 |
| V3 | 6 | 8 |
| V3 | 6 | 10 |
| V3 | 6 | 7 |
| V3 | 6 | 9 |
| V3 | 6 | 9 |
| V3 | 6 | 9 |
| V3 | 6 | 9 |
| K1 | 7 | 9 |
| K1 | 7 | 7 |
| K1 | 7 | 8 |
| K1 | 7 | 9 |
| K1 | 7 | 10 |
| K1 | 7 | 9 |
| K1 | 7 | 8 |
| K1 | 7 | 9 |
| K1 | 7 | 9 |
| K1 | 7 | 8 |
| K1 | 7 | 9 |
| K1 | 7 | 9 |
| K2 | 7 | 8 |
| K2 | 7 | 10 |
| K2 | 7 | 10 |
| K2 | 7 | 9 |
| K2 | 7 | 9 |
| K2 | 7 | 10 |
| K2 | 7 | 7 |
| K2 | 7 | 7 |
| K2 | 7 | 9 |
| K2 | 7 | 11 |
| K2 | 7 | 11 |
| K2 | 7 | 8 |
| V1 | 7 | 9 |
| V1 | 7 | 9 |
| V1 | 7 | 10 |
| V1 | 7 | 10 |
| V1 | 7 | 9 |
| V1 | 7 | 10 |
| V1 | 7 | 11 |
| V1 | 7 | 9 |
| V1 | 7 | 7 |
| V1 | 7 | 7 |
| V1 | 7 | 8 |
| V1 | 7 | 8 |
| V2 | 7 | 8 |
| V2 | 7 | 10 |
| V2 | 7 | 6 |
| V2 | 7 | 8 |
| V2 | 7 | 7 |
| V2 | 7 | 9 |
| V2 | 7 | 8 |
| V2 | 7 | 8 |
| V2 | 7 | 8 |
| V2 | 7 | 8 |
| V2 | 7 | 9 |
| V2 | 7 | 6 |
| V3 | 7 | 7 |
| V3 | 7 | 7 |
| V3 | 7 | 10 |
| V3 | 7 | 11 |
| V3 | 7 | 9 |
| V3 | 7 | 8 |
| V3 | 7 | 8 |
| V3 | 7 | 5 |
| V3 | 7 | 9 |
| V3 | 7 | 10 |
| V3 | 7 | 9 |
| V3 | 7 | 11 |
| K1 | 8 | 10 |
| K1 | 8 | 9 |
| K1 | 8 | 8 |
| K1 | 8 | 9 |
| K1 | 8 | 8 |
| K1 | 8 | 11 |
| K1 | 8 | 9 |
| K1 | 8 | 11 |
| K1 | 8 | 10 |
| K1 | 8 | 9 |
| K1 | 8 | 11 |
| K1 | 8 | 11 |
| K2 | 8 | 8 |
| K2 | 8 | 11 |
| K2 | 8 | 8 |
| K2 | 8 | 9 |
| K2 | 8 | 8 |
| K2 | 8 | 11 |
| K2 | 8 | 8 |
| K2 | 8 | 11 |
| K2 | 8 | 7 |
| K2 | 8 | 9 |
| K2 | 8 | 9 |
| K2 | 8 | 8 |
| V1 | 8 | 10 |
| V1 | 8 | 8 |
| V1 | 8 | 5 |
| V1 | 8 | 5 |
| V1 | 8 | 9 |
| V1 | 8 | 10 |
| V1 | 8 | 10 |
| V1 | 8 | 9 |
| V1 | 8 | 11 |
| V1 | 8 | 8 |
| V1 | 8 | 6 |
| V1 | 8 | 5 |
| V2 | 8 | 9 |
| V2 | 8 | 8 |
| V2 | 8 | 9 |
| V2 | 8 | 9 |
| V2 | 8 | 9 |
| V2 | 8 | 9 |
| V2 | 8 | 10 |
| V2 | 8 | 10 |
| V2 | 8 | 9 |
| V2 | 8 | 8 |
| V2 | 8 | 8 |
| V2 | 8 | 10 |
| V3 | 8 | 10 |
| V3 | 8 | 8 |
| V3 | 8 | 10 |
| V3 | 8 | 10 |
| V3 | 8 | 8 |
| V3 | 8 | 10 |
| V3 | 8 | 11 |
| V3 | 8 | 8 |
| V3 | 8 | 10 |
| V3 | 8 | 8 |
| V3 | 8 | 7 |
| V3 | 8 | 8 |
| K1 | 9 | 8 |
| K1 | 9 | 9 |
| K1 | 9 | 9 |
| K1 | 9 | 9 |
| K1 | 9 | 9 |
| K1 | 9 | 7 |
| K1 | 9 | 8 |
| K1 | 9 | 9 |
| K1 | 9 | 8 |
| K1 | 9 | 9 |
| K1 | 9 | 9 |
| K1 | 9 | 7 |
| K2 | 9 | 7 |
| K2 | 9 | 8 |
| K2 | 9 | 9 |
| K2 | 9 | 9 |
| K2 | 9 | 8 |
| K2 | 9 | 10 |
| K2 | 9 | 9 |
| K2 | 9 | 10 |
| K2 | 9 | 10 |
| K2 | 9 | 10 |
| V1 | 9 | 8 |
| V1 | 9 | 7 |
| V1 | 9 | 11 |
| V1 | 9 | 9 |
| V1 | 9 | 11 |
| V1 | 9 | 9 |
| V1 | 9 | 9 |
| V1 | 9 | 9 |
| V1 | 9 | 11 |
| V1 | 9 | 11 |
| V1 | 9 | 9 |
| V1 | 9 | 9 |
| V2 | 9 | 10 |
| V2 | 9 | 9 |
| V2 | 9 | 9 |
| V2 | 9 | 8 |
| V2 | 9 | 9 |
| V2 | 9 | 8 |
| V2 | 9 | 8 |
| V2 | 9 | 9 |
| V2 | 9 | 8 |
| V2 | 9 | 8 |
| V2 | 9 | 9 |
| V2 | 9 | 10 |
| V3 | 9 | 9 |
| V3 | 9 | 8 |
| V3 | 9 | 8 |
| V3 | 9 | 10 |
| V3 | 9 | 8 |
| V3 | 9 | 10 |
| V3 | 9 | 8 |
| V3 | 9 | 11 |
| V3 | 9 | 9 |
| V3 | 9 | 10 |
| V3 | 9 | 9 |
| V3 | 9 | 10 |
